# Wireless power transfer system with enhanced efficiency by using frequency reconfigurable metamaterial

**DOI:** 10.1038/s41598-021-03570-8

**Published:** 2022-01-10

**Authors:** Dongyong Shan, Haiyue Wang, Ke Cao, Junhua Zhang

**Affiliations:** 1 Postdoctoral Research Station of Clinical Medicine and Department of Oncology Radiotherapy Center, The 3Rd Xiangya Hospital, Central South University, Changsha, 410000 China; 2 Department of Blood Transfusion, The Third Xiangya Hospital of Central South University, Changsha, 410013 China; 3 School of Physics and Electronics, Central South University, Changsha, 410083 China

**Keywords:** Electrical and electronic engineering, Power distribution, Electronic devices, Devices for energy harvesting

## Abstract

The wireless power transfer (WPT) system has been widely used in various fields such as household appliances, electric vehicle charging and sensor applications. A frequency reconfigurable magnetic resonant coupling wireless power transfer (MRCWPT) system with dynamically enhanced efficiency by using the frequency reconfigurable metamaterial is proposed in this paper. The reconfigurability is achieved by adjusting the capacitance value of the adjustable capacitor connected in the coil of the system. Finite element simulation results have shown that the frequency reconfigurable electromagnetic metamaterial can manipulate the direction of the electromagnetic field of the system due to its abnormal effective permeability. The ultra-thin frequency reconfigurable metamaterial is designed at different working frequencies of 14.1 MHz, 15 MHz, 16.2 MHz, 17.5 MHz, 19.3 MHz, 21.7 MHz and 25 MHz to enhance the magnetic field and power transfer efficiency (PTE) of the system. Frequency reconfigurable mechanism of the system with the frequency reconfigurable metamaterial is derived by the equivalent circuit theory. Finally, further measurement which verifies the simulation by reasonable agreement is carried out. PTE of the system by adding the metamaterial are 59%, 73%, 67%, 66%, 65%, 60% and 58% at different working frequencies. PTE of the system with and without the metamaterial is 72% and 49% at the distance of 120 mm and the frequency of 15 MHz, respectively.

## Introduction

The wireless power transfer (WPT) system is used for the transmission of energy without a direct physical cable connection, which is useful to power loads where using cables is hazardous and inconvenient^1–3^. The magnetic resonant coupling wireless power transfer (MRCWPT) system has relatively high transfer efficiency over relatively long distances, and the MRCWPT system has gained lots of attention. And the MRCWPT system is much promising in the field of charging^4–6^, which has been applied in potential applications such as medical implants, electric vehicle charging, sensor networks and consumer electronics^7–9^.

For conventional MRCWPT systems, both the transmitter and the receiver have the same resonant frequency to maintain relatively high power transfer efficiency (PTE)^10,11^. The receiver and the transmitter work at the single resonant frequency. When the electrical energy is transmitted from the transmitter to the receiver at the different working frequency, failures and malfunctions may be caused regardless of receivers’ demands. Also, PTE of the system reduces. In order to solve the problem, the frequency reconfigurable MRCWPT system with additional control circuits is proposed in^12^ by changing the resonant capacitance value. An efficient and reconfigurable rectifier circuit, with the capability of automatically switching from low-power to high-power operation mode, is presented in^13^. The new topology allows the rectifier to convert RF power to DC power efficiently over an extended input power range. The frequency reconfigurable technology is achieved in^14^ by varying the distance between the receiver and the transmitter of the MRCWPT system. A shape-reconfigurable MRCWPT system in^15^ achieves frequency reconfigurability by different structures of resonant coils. A novel planar-spiral transmitter coil (TX-coil) with an outer-tight and inner-sparse configuration is proposed to achieve a high quality factor and uniform magnetic field, which ensures high efficiency and improves the misalignment tolerance for several-megahertz WPT systems in^16^. The above MRCWPT systems have the frequency reconfigurable property, but volume and complexity of the system increase. In order to obtain higher PTE and power receivers at different frequencies, a frequency reconfigurable MRCWPT system is presented by adjusting the capacitance value of the adjustable capacitor connected in the coil of the system in this paper.

At present, many researchers proposed MRCWPT systems to further enhance PTE and extend the distance of the system. A kind of method by adding relay resonators is proposed in^17^. It is obvious that the distance and PTE of the system are extended. Intermediate resonators arranged between the transmitter and the receiver are used to transmit the magnetic field. This method is used to improve PTE of the system to maximize the benefits of magnetic field repeaters^18^. In^19–21^, MRCWPT systems with the metamaterial are proposed. Some MRCWPT systems with repeaters and metamaterial are analyzed for applications in^22^. The analysis shows that PTE of MRCWPT systems with repeaters and metamaterial is improved in different ways. Metamaterial can provide the MRCWPT system with various tunable functions. And the MRCWPT system with nonidentical coils using metamaterial is proposed in^21^. However, further investigation should be carried out about MRCWPT systems using metamaterial to improve the PTE and the distance. Investigations about the metamaterial is mainly on the far field, but the metamaterial used in the MRCWPT system on near field is lacking. Recently, some MRCWPT systems using metamateial are reported in^23–27^. Theoretical analysis and experimental investigation about using metamaterial to improve PTE of the system are shown. In^23^, PTE of the system increases from 17 to 35% by using metamaterial at the working frequency of 27.12 MHz. Maximum 25.4% efficiency enhancement is achieved when the distance between Tx and Rx coils is 15 cm, and in overall distance variation cases, the proposed two-stack hybrid metamaterial slab make the power transfer efficiency increase in^26^. The metamaterial is used in the MRCWPT system, and the enhanced PTE is 54.3% at the distance of 1.0 m in^27^. The performance of the MRCWPT system is improved by using metamaterial in the above work. However, the metamaterial is so thick and large that it limits the application of the system. The conventional metamaterial used for the MRCWPT system to improve the efficiency just works at only single frequency. Also, the research about metamaterial used for frequency reconfigurable magnetic resonant coupling wireless power transfer system is lacking. This paper presents a method for improving the efficiency of the frequency reconfigurable wireless power transfer system dynamically by using the frequency reconfigurable metamaterial at the different working frequency. The reconfigurability is achieved by adjusting the capacitance value of the adjustable capacitor connected in the coil of the system. The conventional structures of the coil and the metamaterial are used in the system, so the universality of this method is further illustrated.

This paper is organized as follows. The theoretical analysis of the frequency reconfigurable wireless power transfer system is illustrated in “Theoretical analysis of the frequency reconfigurable wireless power transfer system” section. Frequency reconfigurable mechanism of the system with the frequency reconfigurable metamaterial is derived by the equivalent circuit theory. Simulation of the frequency reconfigurable wireless power transfer system is presented in “Simulation of the frequency reconfigurable wireless power transfer system” section. The experimental results compared with simulation results of the MRCWPT system are illustrated in “Measurement of the frequency reconfigurable wireless power transfer system” section. Finally, conclusions are drawn in “Conclusion” section.

## Theoretical analysis of the frequency reconfigurable wireless power transfer system

**Figure 1:**
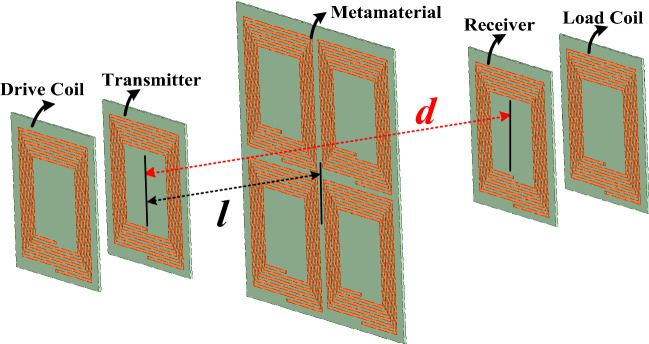
The frequency reconfigurable MRCWPT system with the frequency reconfigurable metamaterial.

The frequency reconfigurable wireless power transfer system with the frequency reconfigurable metamaterial is shown in Fig. 1. The system includes a drive coil, a transmitter, a receiver, and a load coil. All the coil structures are square spiral coils because of simple fabrication and popular applications. The energy is transmitted from the transmitter to the receiver through the electromagnetic field. The metamaterial shown in Fig. 1 is used to manipulate the direction of the electromagnetic field of the system to enhance the PTE of the system. The variable *d* represents the distance between the receiver and the transmitter. The variable *l* represents the distance between the transmitter and the metamaterial. When the reconfigurable metamaterial works at the different frequency, the drive loop, the transmitter, the receiver, and the load loop need to be working at the same frequency with the metamaterial. The control method and strategy of the system is described as follows. The frequency reconfigurable MRCWPT system is achieved by adjusting the capacitance value of the adjustable capacitor (TZY2K450A001) connected in the coil of the system in this paper. There is a adjustable capacitor connected in the the drive loop, the transmitter, the receiver, and the load loop, respectively. And four identical adjustable capacitors connected in four coils of the metamaterial are used to achieve the frequency reconfigurability of the metamaterial by changing the capacitance value. The capacitance value of the adjustable capacitor is changed from 8 to 45 pf by adjusting the distance between the plate and the fixed plate with screws. When the reconfigurable metamaterial works at the different frequency by changing the capacitance value of the adjustable capacitor (TZY2K450A001), the drive loop, the transmitter, the receiver, and the load loop need to be working at the same frequency with the metamaterial by changing the capacitance value of the identical adjustable capacitor (TZY2K450A001).

**Figure 2:**
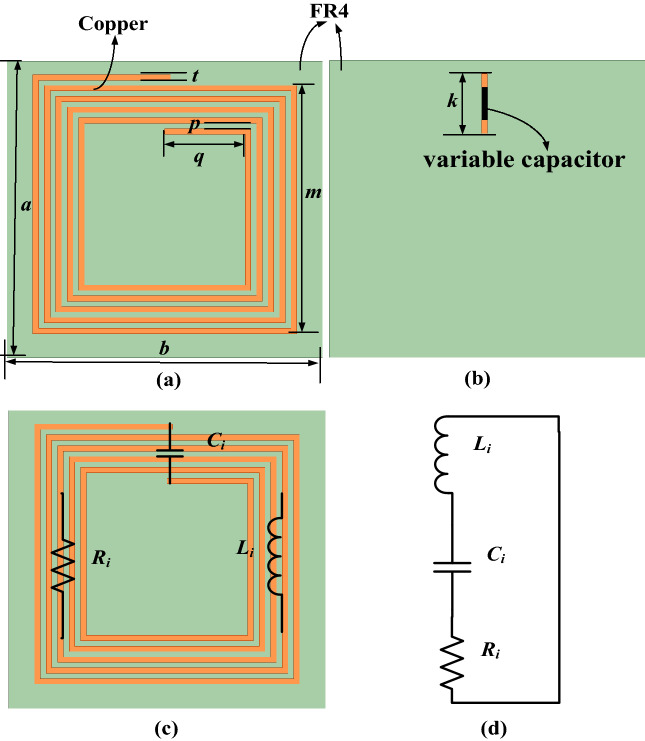
(**a**) Top and (**b**) bottom view of the coil unit cell, (**c**) the circuit schematic of the coil unit cell, (**d**) the equivalent RLC resonant circuit of the coil structure.

In order to clarify the proposed frequency reconfigurable MRCWPT system with the frequency reconfigurable metamaterial clearly by using the equivalent lumped circuit model, the schematic structure of the coil unit cell can be modeled into the RLC resonant circuit. Top and bottom view of the coil unit cell are shown in Fig. 2a and b, respectively. The circuit schematic of the coil unit cell is illustrated in Fig. 2c, and the equivalent RLC resonant circuit of the coil structure is shown in Fig. 2d. The equivalent inductance of the copper coil is *L*_*i*_, and the equivalent capacitance of the copper coil is *C*_*i*_. *R*_*i*_ is the equivalent resistance in the equivalent RLC resonant circuit. When the capacitance value of the variable capacitor in Fig. 2b changes, *C*_*i*_ will change accordingly. The concrete dimension of the coil unit shown in Fig. 2 is illustrated in Table 1.

**Table 1:** The dimension of the coil unit.

Parameters	Value (mm)
*a*	110
*b*	110
*t*	2
*m*	92
*p*	2
*q*	28
*k*	20

**Figure 3:**
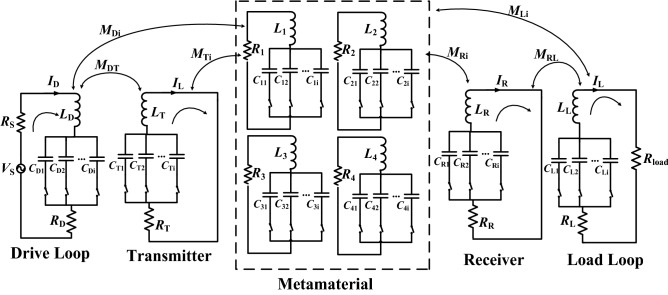
The equivalent circuit of the frequency reconfigurable wireless power transfer system with the frequency reconfigurable metamaterial.

The frequency reconfigurable MRCWPT system with the frequency reconfigurable metamaterial is modeled by using the equivalent circuit theory. The equivalent circuit of the frequency reconfigurable system is presented in Fig. 3. There are eight equivalent circuits corresponding to eight coil unit cells in the frequency reconfigurable MRCWPT system with the frequency reconfigurable metamaterial. *I*_*i*_ and *Z*_*i*_ (*i* = 1, 2, …, 4) represent the current and impedance of the coil unit cell of the frequency reconfigurable metamaterial. *I*_D_, *I*_R_, *I*_T_, and *I*_L_ represent the current of the drive coil, the receiver, the transmitter, and the load loop, respectively. *Z*_D_, *Z*_R_, *Z*_T_, and *Z*_L_ are impedance of the drive coil, the receiver, the transmitter, and the load loop, respectively. *L*_D_, *L*_T_, *L*_R_, *L*_L_ and *L*_*i*_ are equivalent inductance. *R*_D_, *R*_T_, *R*_R_, *R*_L_ and *R*_i_ are equivalent resistances. *C*_D_, *C*_T_, *C*_R_,*C*_L_ and *C*_*i*_ are equivalent capacitance. The drive coil and the transmitter is connected by the mutual inductance *M*_DT_. The transmitter and the coil unit cell of the metamaterial is connected by the mutual inductance *M*_T*i*_. The drive coil and the coil unit cell of the metamaterial is connected by the mutual inductance *M*_D*i*_. The coil unit cell of the metamaterial and the receiver is connected by the mutual inductance *M*_R*i*_. The coil unit cell of the metamaterial and the load coil is connected by the mutual inductance *M*_Li_. The load coil and the receiver is connected by the mutual inductance *M*_RL_. In Fig. 3, the source has the voltage of *V*_S_ and impedance of *R*_S_. *R*_load_ is connected with the load coil. The variable *w* represents the resonant frequency of the system.

According to the Kirchhoff’s voltage law and the mutual coupling theory, equations of voltages and currents can be shown as follows:

1 $$ \left[ \begin{gathered} V_{S} \\ 0 \\ 0 \\ \vdots \\ 0 \\ \end{gathered} \right] = \left[ \begin{gathered} R_{S} + Z_{D} \, jwM_{DT} {\text{ jwM}}_{{{\text{D1}}}} \, \cdots {\text{jwM}}_{{{\text{DL}}}} \hfill \\ jwM_{DT} {\text{ Z}}_{{\text{T}}} {\text{ jwM}}_{{{\text{T1}}}} \, \cdots {\text{jwM}}_{{{\text{TL}}}} \hfill \\ jwM_{D1} {\text{ jwM}}_{{{\text{T1}}}} {\text{ Z}}_{{1}} \, \cdots {\text{jwM}}_{{{\text{1L}}}} \hfill \\ \, \vdots \, \vdots \, \vdots \, \vdots \hfill \\ jwM_{DL} {\text{ jwM}}_{{{\text{TL}}}} {\text{ jwM}}_{{{\text{1L}}}} \cdots {\text{R}}_{L} + Z_{L} \hfill \\ \end{gathered} \right]\left[ \begin{gathered} I_{D} \\ I_{T} \\ I_{1} \\ \vdots \\ I_{L} \\ \end{gathered} \right] $$

where

2 $$ \left\{ \begin{gathered} Z_{D} = R_{D} + {\text{j(}}wL_{D} - \frac{1}{{wC_{{D{\text{i}}}} }}) \hfill \\ Z_{T} = R_{T} + {\text{j(}}wL_{T} - \frac{1}{{wC_{Ti} }}) \hfill \\ Z_{i} = R_{i} + {\text{j(}}wL_{i} - \frac{1}{{wC_{ii} }}) \hfill \\ Z_{R} = R_{R} + {\text{j(}}wL_{R} - \frac{1}{{wC_{Ri} }}) \hfill \\ Z_{L} = R_{L} + {\text{j(}}wL_{L} - \frac{1}{{wC_{Li} }}) \hfill \\ \end{gathered} \right. $$

3 $$ S_{21} = 2\frac{{V_{L} }}{{V_{S} }}\sqrt {\frac{{R_{S} }}{{R_{L} }}} $$

The PTE of the system can be calculated by using the scattering parameter |*S*_21_| by PTE =|*S*_21_|^2^.

## Simulation of the frequency reconfigurable wireless power transfer system

### The frequency reconfigurable metamaterial

**Figure 4:**
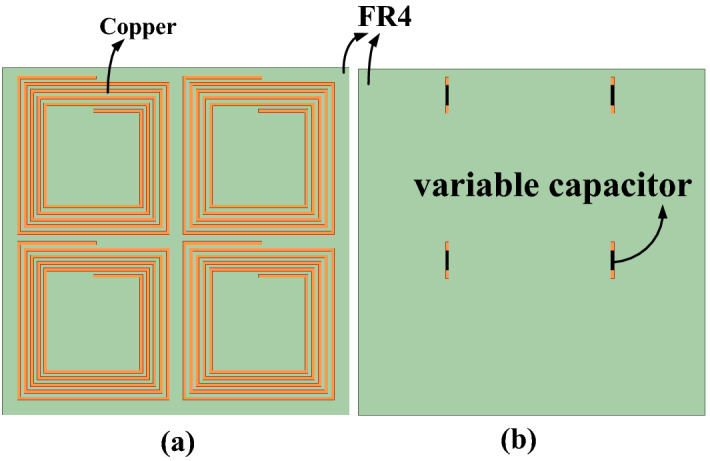
(**a**) Top and (**b**) bottom view of the frequency reconfigurable metamaterial.

The frequency reconfigurable metamaterial is used to enhance PTE of the frequency reconfigurable wireless power transfer system. Top and bottom view of the frequency reconfigurable metamaterial are shown in Fig. 4a and b, respectively. There are four copper coil unit cells on the FR4 dielectric substrate. And four identical adjustable capacitors connected in four coils are used to achieve the reconfigurability of the metamaterial by changing their capacitance value.

**Figure 5:**
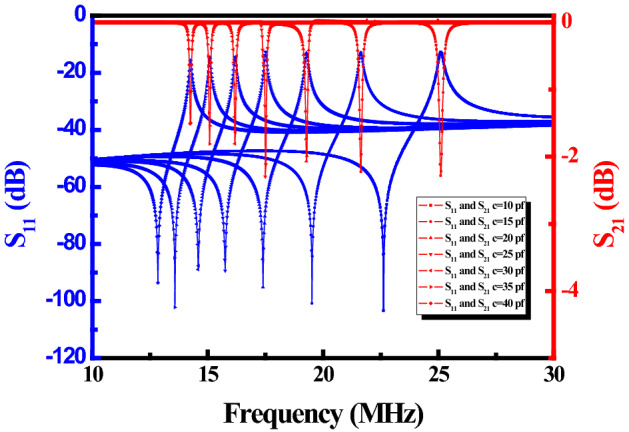
The reflection loss *S*_11_ and transmission coefficient *S*_21_ of the frequency reconfigurable metamaterial at different frequencies.

By changing the capacitance value of the adjustable capacitor, the reflection loss *S*_11_ and the transmission coefficient *S*_21_ of the frequency reconfigurable metamaterial at different frequencies are illustrated in Fig. 5. The parameters of the reflection loss *S*_11_ and transmission coefficient *S*_21_ changes dramatically at different frequency bands, which indicates that the metamaterial has some special electromagnetic characteristics at this frequency band. The ultra-thin frequency reconfigurable metamaterial is designed at the different working frequency of 14.1 MHz, 15 MHz, 16.2 MHz, 17.5 MHz, 19.3 MHz, 21.7 MHz and 25 MHz to enhance the magnetic field and PTE of the system.

The general approach to the retrieval of material parameters from *S* parameters for homogeneous materials is outlined as follows. For the sake of generality, it is useful to first define the one-dimensional transfer matrix, which relates the fields on one side of a planar slab to the other. The transfer matrix can be defined from

4 $$ F^{\prime} = TF $$

where

5 $$ F = \left( {\begin{array}{*{20}c} E \\ {H_{red} } \\ \end{array} } \right) $$

*E* and *H*_red_ are the complex electric and magnetic field amplitudes located on the right-hand and left-hand faces of the slab. Note that the magnetic field assumed throughout is a reduced magnetic field having the normalization $H_{red} = ( + i{\text{w}}\mu_{0} )H$. The transfer matrix for a homogeneous 1D slab has the analytic form.

6 $$ T = \left( {\begin{array}{*{20}c} {\cos (nkd)} & { - \frac{k}{z}\sin (nkd)} \\ {\frac{k}{z}\sin (nkd)} & {\cos (nkd)} \\ \end{array} } \right) $$

where n is the refractive index and z is the wave impedance of the slab. n and z are related to the effective permeability μ by the relation

7 $$ \mu = nz $$

The total field amplitudes are not conveniently probed in measurements, whereas the scattered field amplitudes and phases can be measured in a straightforward manner. A scattering S matrix relates the incoming field amplitudes to the outgoing field amplitudes, and can be directly related to experimentally determined quantities. The elements of the S matrix can be found from the elements of the T matrix as follows:

8 $$ S_{21} = \frac{2}{{T_{11} + T_{22} + (ikT_{12} + \frac{{T_{21} }}{ik})}} $$

9 $$ S_{11} = \frac{{T_{11} - T_{22} + ({\text{ik}}T_{12} - \frac{{T_{21} }}{ik})}}{{T_{11} + T_{22} + ({\text{ik}}T_{12} + \frac{{T_{21} }}{ik})}} $$

10 $$ S_{22} = \frac{{T_{22} - T_{11} + (ikT_{12} - \frac{{T_{21} }}{ik})}}{{T_{22} + T_{11} + (ikT_{12} + \frac{{T_{21} }}{ik})}} $$

11 $$ S_{12} = \frac{2\det (T)}{{T_{22} + T_{11} + (ikT_{12} + \frac{{T_{21} }}{ik})}} $$

For a slab of homogeneous material, T11=T22=Ts and det (T)=1, and the S matrix is symmetric. Thus,

12 $$ S_{12} = S_{21} = \frac{1}{{T_{S} + \frac{1}{2}(ikT_{12} + \frac{{T_{21} }}{ik})}} $$

13 $$ S_{11} = S_{22} = \frac{{\frac{1}{2}(\frac{{T_{21} }}{ik} - ikT_{12} )}}{{T_{S} + \frac{1}{2}(ikT_{12} + \frac{{T_{21} }}{ik})}} $$

Using the analytic expression for the *T*-matrix elements gives the *S* parameter

14 $$ S_{21} = S_{12} = \frac{1}{{\cos (nkd) - \frac{i}{2}(z + \frac{1}{z})\sin (nkd)}} $$

15 $$ S_{11} = S_{22} = \frac{i}{2}(\frac{1}{z} - z)\sin (nkd) $$

Equations can be inverted to find n and z in terms of the scattering parameters as follows:

16 $$ n = \frac{1}{kd}\cos^{ - 1} \left[ {\frac{1}{{2S_{21} }}(1 - S_{11}^{2} - S_{21}^{2} )} \right] $$

17 $$ z = \sqrt {\frac{{(1 + S_{11} )^{2} - S_{21}^{2} }}{{(1 - S_{11} )^{2} - S_{21}^{2} }}} $$

Therefore, the effective permeability can be obtained with *S*_11_ and *S*_21_.

**Figure 6:**
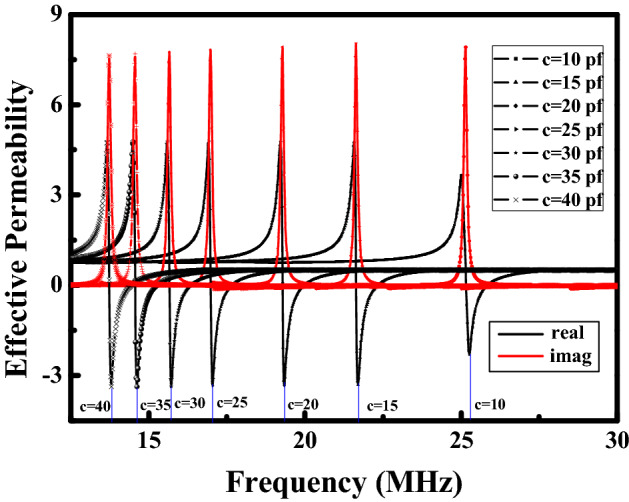
The effective permeability of the frequency reconfigurable metamaterial at different frequencies.

The parameter extraction method is adopted to calculate the effective permeability of the frequency reconfigurable metamaterial according to the reflection loss *S*_11_ and the transmission coefficient *S*_21_ in Fig. 5. The effective permeability of the frequency reconfigurable metamaterial at different frequencies is shown in Fig. 6. The red curve in Fig. 6 represents the imaginary part of the effective permeability. The black curve represent the real part of the effective permeability. The effective permeability of the frequency reconfigurable metamaterial is negative at the different resonant frequency.

### The location optimization of the frequency reconfigurable metamaterial

**Figure 7:**
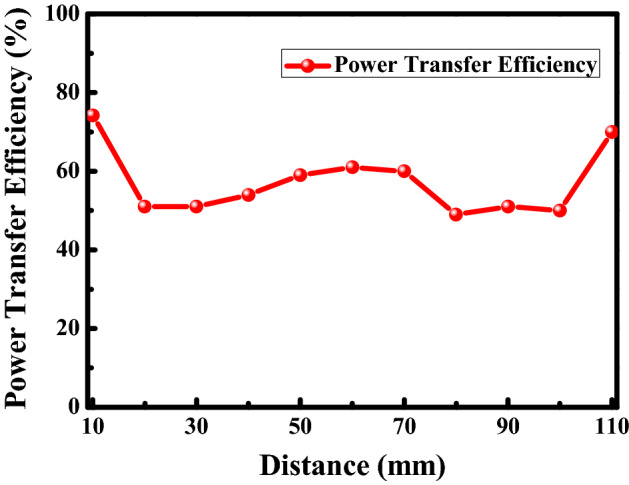
The PTE of the system at the different distance between the transmitter and the metamaterial.

The frequency reconfigurable metamaterial is located between the transmitter and the receiver as shown in Fig. 1. The distance between the metamaterial and the transmitter is *l*. In order to enhance PTE of the wireless power transfer system, the distance *l* between the transmitter and the metamaterial should be optimized. The PTE of the wireless power transfer system at the different distance between the transmitter and the metamaterial is presented in Fig. 7. The distance d between the transmitter and the receiver is 120 mm. The distance *l* between the transmitter and the metamaterial varies from 10 to 110 mm. When the distance *l* is 110 mm, the distance between the metamaterial and the receiver is 10 mm. Due to the system symmetry, it can be seen that the efficiency of the system at 10 mm is high and also at 110 mm distance is high. It is shown that the PTE is 77% at the distance of 10 mm and 110 mm. And the PTE at the distance is higher than the PTE at the other different distance. Therefore, the optimized distance between the transmitter and the metamaterial is 10 mm.

### The magnetic field distribution of the system

**Figure 8:**
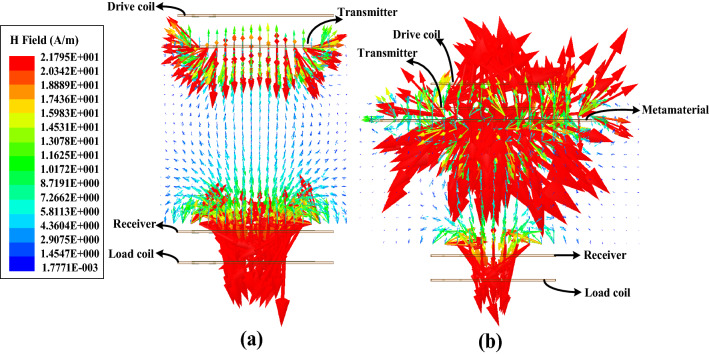
(**a**) The magnetic field distribution of the system without the metamaterial, (**b**) The magnetic field distribution of the system with the metamaterial.

The magnetic field distribution of the system without the metamaterial and the magnetic field distribution of the system with the metamaterial are presented in Fig. 8a and b, respectively. The magnetic field distribution of the system is shown to better understand the coupling between the transmitter and the receiver. It is shown that the coupling between the transmitter and the receiver with the metamaterial is better than the coupling between the transmitter and the receiver without the metamaterial. Therefore, the metamaterial can be used to manipulate the direction of the electromagnetic field of the system due to its abnormal effective permeability.

## Measurement of the frequency reconfigurable wireless power transfer system

**Figure 9:**
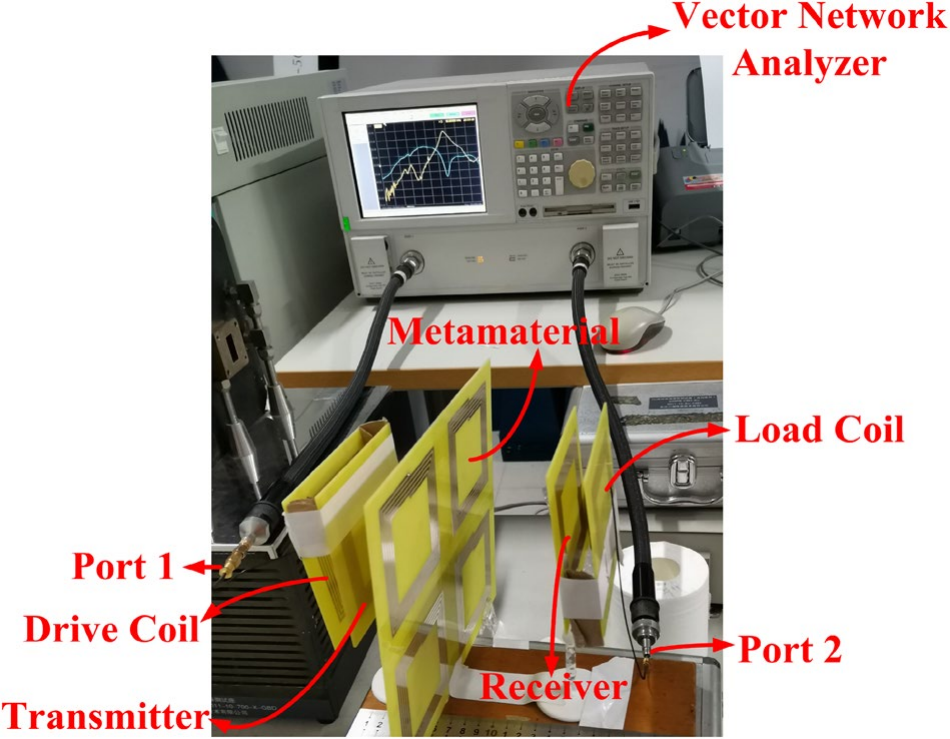
The experimental platform of the frequency reconfigurable MRCWPT system.

The coils and the frequency reconfigurable metamaterial are fabricated by using parameters shown in Table 2 to verify the correctness of the simulation results of the frequency reconfigurable wireless power transfer system. The experimental platform of the frequency reconfigurable wireless power transfer system is presented in Fig. 9. The drive coil and the load coil are connected to the port 1 and the port 2 of the vector network analyzer (Agilent Technologies N5230A), respectively. The drive coil is fixed in a certain position, and the transmitter, the frequency reconfigurable metamaterial, the receiver and the load coil are placed in a coaxial position of the load coil. PTE of the MRCWPT system is calculated by using the equation in^29^

$$ PTE = \left| {S_{{{21}}} } \right|^{2} \times {\text{100\% }} $$

**Table 2:** The Parameters of the coil unit.

Parameters	Value
Structure	Plane
Material	Copper
Wire diameter	2 mm
Wire spacing	2 mm
Turn	4
Coil diameter	92 mm

**Figure 10:**
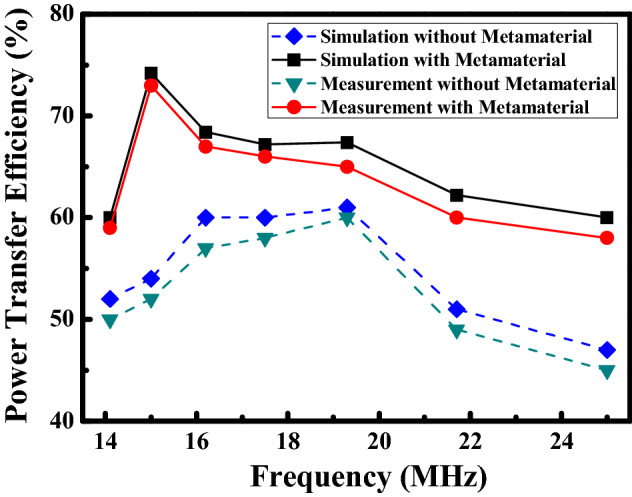
Simulated and measured PTE at the different frequency with and without metamaterial.

To verify the enhancement of the MRCWPT system with the frequency reconfigurable metamaterial, the MRCWPT system with and without the frequency reconfigurable metamaterial are carried out by the experiments, respectively. PTE of the MRCWPT system at the different frequency with and without the frequency reconfigurable metamaterial are presented in Fig. 10. Obviously, PTE of the MRCWPT system with the frequency reconfigurable metamaterial is higher than PTE of the system without the metamaterial. PTE of the system with the frequency reconfigurable metamaterial are 59%, 73%, 67%, 66%, 65%, 60% and 58% at different frequencies of 14.1 MHz, 15 MHz, 16.2 MHz, 17.5 MHz, 19.3 MHz, 21.7 MHz and 25 MHz, respectively.

**Figure 11:**
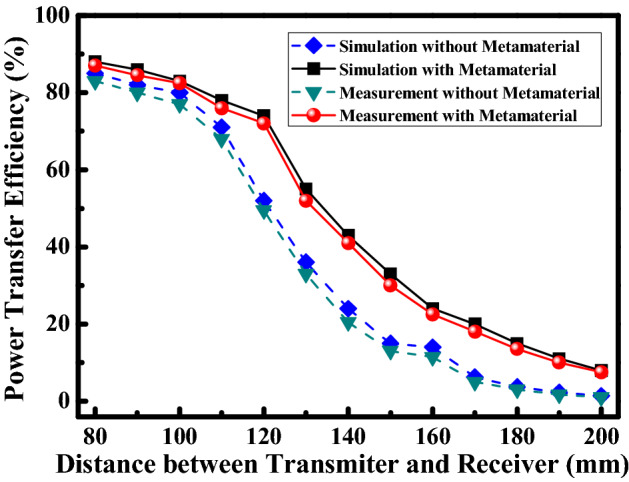
PTE of the wireless power transfer system at the different transmission distance with and without metamaterial.

Simulated and measured PTE of the wireless power transfer system at the different transmission distance with and without the metamaterial are presented in Fig. 11. Measurement results of the system agree well with simulation results. The deviation may be due to the practical fabrication and measurement errors. The PTE of the wireless power transfer system is improved obviously at the different transmission distance by using the metamaterial. The results indicate that high PTE of the MRCWPT system is carried out by using the metamaterial as the magnetic flux guide. PTE of the system with and without the metamaterial is 72% and 49% at the distance of 120 mm and the frequency of 15 MHz, respectively. Experiment results intuitively verify that PTE of the MRCWPT system is indeed enhanced significantly by using the metamaterial.

## Conclusion

A frequency reconfigurable MRCWPT system with enhanced efficiency by using the frequency reconfigurable metamaterial is presented in this paper. Frequency reconfigurable mechanism of the system with the frequency reconfigurable metamaterial is derived by the equivalent circuit theory. Finite element simulation results have shown that the frequency reconfigurable electromagnetic metamaterial can manipulate the direction of the electromagnetic field of the system due to its abnormal effective permeability. The location optimization of the frequency reconfigurable metamaterial shows that the optimized distance between the transmitter and the metamaterial is 10 mm. Further measurement which verifies the simulation by reasonable agreement is carried out. PTE of the system by adding the frequency reconfigurable metamaterial are 59%, 73%, 67%, 66%, 65%, 60% and 58% at different working frequencies of 14.1 MHz, 15 MHz, 16.2 MHz, 17.5 MHz, 19.3 MHz, 21.7 MHz and 25 MHz, respectively. PTE of the system with and without the metamaterial is 72% and 49% at the distance of 120 mm and the frequency of 15 MHz, respectively. PTE is improved obviously at the different frequency and the different transmission distance by using the frequency reconfigurable metamaterial.

## References

[1] Li, Y., Zhang, L., Zhao, T., Zou, L., The electromagnetic compatibility analysis of experimental apparatus based on wireless power transmission. *IEEE Indust. Electron. Appl.*, pp 2334–2338, (2016).

[2] Kuo N-C, Zhao B, Niknejad AM (2016). Bifurcation analysis in weakly coupled inductive power transfer systems. IEEE Trans. Circuits Syst. I Reg. Papers.

[3] Zhang Z, Pang H, Georgiadis A, Cecati C (2019). Wireless power transfer—An overview. IEEE Trans. Indust. Electron..

[4] Zhang C, Chen Y (2017). ‘Wireless power transfer strategies for cooperative relay system to maximize information throughput’. IEEE Access.

[5] Hoang H, Lee S, Kim Y, Choi Y, Bien F (2012). An adaptive technique to improve wireless power transfer for consumer electronics. IEEE Trans. Consum. Electron..

[6] Kurs A, Karalis A, Moffatt R, Joannopoulos JD, Fisher P, Soljacic M (2007). Wireless power transfer via strongly coupled magnetic resonances. Science.

[7] Basar MR, Ahmadm MY, Cho J, Ibrahim F (2017). ‘Stable and high efficiency wireless power transfer system for robotic capsule using a modified helmholtz coil’. IEEE Trans. Ind. Electron..

[8] Yedavalli PS, Riihonen T, Wang X, Rabaey JM (2017). ‘Far-field RF wireless power transfer with blind adaptive beam forming for Internet of Things devices’. IEEE Access.

[9] Monti G, Che W, Wang Q, Costanzo A, Chang Y (2017). Wireless power transfer with three-ports networks: Optimal analytical solutions. IEEE Trans. Circuits Syst. I Reg. Papers.

[10] Choi, J., Cho, J. K., and Seo, C., Analysis on transmission efficiency of wireless energy transmission resonator based on magnetic resonance. *IEEE Microwave Workshop Ser. Innovat. Wireless Power Trans. Technol.*, pp. 199–202, (2011).

[11] Kim J-M, Han M, Sohn H (2015). Magnetic resonance-based wireless power transmission through concrete structures. J. Electromagn. Eng. Sci..

[12] Ngo T, Huang A, Guo Y (2019). Analysis and design of a reconfigurable rectifier circuit for wireless power transfer. IEEE Trans. Industr. Electron..

[13] Dai Z, Fang Z, Huang H, He Y, Wang J (2018). Selective omnidirectional magnetic resonant coupling wireless power transfer with multiple-receiver system. IEEE Access.

[14] Duong TP, Lee JW (2011). Experimental results of high-efficiency resonant coupling wireless power transfer using a variable coupling method. IEEE Microw. Wireless Compon. Lett..

[15] Liu Z, Chen Z, Li J, Zhao H (2016). A shape-reconfigurable modularized wireless power transfer array system for multipurpose wireless charging applications. IEEE Trans. Antennas Propag..

[16] Wu L, Zhang B (2019). Reconfigurable transmitter coil structure for highly efficient and misalignment-insensitive wireless power transfer systems in megahertz range. Chin. J. Electr. Eng..

[17] Lee W, Yoon Y-K (2020). Tunable metamaterial slab for efficiency improvement in misaligned wireless power transfer. IEEE Microwave Wirel. Compon. Lett..

[18] Ahn D, Hong S (2013). A study on magnetic field repeater in wireless power transfer. IEEE Trans. Ind. Electron..

[19] Wang H, Wang W, Chen X, Li Q, Zhang Z (2020). Analysis and design of khz-metamaterial for wireless power transfer. IEEE Trans. Magn..

[20] Liu, J., Chen, Z., Zhou, J. and Sun, H., Compact triplex-layer metamaterials design for wireless power transfer efficiency enhancement. 2020 IEEE 19th Biennial Conference on Electromagnetic Field Computation (CEFC), 1–4, (2020).

[21] Pham TS, Ranaweera AK, Lam VD, Lee JW (2016). Experiments on localized wireless power transmission using a magneto-inductive wave two-dimensional metamaterial cavity. Appl. Phys. Exp..

[22] Chabalko MJ, Besnoff J, Ricketts DS (2016). Magnetic field enhancement in wireless power with metamaterials and magnetic resonant couplers. IEEE Antenna Wireless Propag. Lett..

[23] Wang B, Teo KH, Nishino T, Yerazunis W, Barnwell J, Zhang J (2011). Experiments on wireless power transfer with metamaterials. Appl. Phys. Lett..

[24] Jia, L. and Fujimori, K. (2021) Improvement of transmission efficiency by using annular array metamaterial for magnetic coupling wireless power transmission system. 2020 International Symposium on Antennas and Propagation (ISAP), pp. 735–736, (2021).

[25] Wang B, Yerazunis W, Teo KH (2013). Wireless power transfer: Metamaterials and array of coupled resonators. Proc. IEEE.

[26] Lee, S. et al., High efficiency wireless power transfer system using a two-stack hybrid metamaterial slab. 2019 IEEE Wireless Power Transfer Conference (WPTC), pp. 616–619, (2019).

[27] Ranaweera ALAK, Doung TP, Lee JW (2014). Experimental investigation of compact metamaterial for high efficiency midrange wireless power transfer applications. J. Appl. Phys..

[28] Ranaweera ALAK, Moscoso CA, Lee JW (2015). Anisotropic metamaterial for efficiency enhancement of mid-range wireless power transfer under coil misalignment. J. Phys. D Appl. Phys..

[29] Sample AP, Meyer DA, Smith JR (2011). Analysis, experimental results, and range adaptation of magnetically coupled resonators for wireless power transfer. IEEE Trans. Ind. Electron..

